# Supplementary material for: Near-field infrared microscopy of nanometer-sized nickel clusters inside single-walled carbon nanotubes
Source: RSC Adv. 2019 Oct 23;9(59):34120–4. doi: 10.1039/c9ra07089c (PMC9073632; doi:10.1039/c9ra07089c)
Supplement: RA-009-C9RA07089C-s001 [file RA-009-C9RA07089C-s001.pdf]

Electronic Supplementary Material (ESI) for RSC Advances.
This journal is © The Royal Society of Chemistry 2019

Supporting Information

Near-field optical investigation of Ni clusters inside single-walled carbon nanotubes on the nanometer scale

Gergely Németh[1], Dániel Datz[1], Áron Pekker[*1], Takeshi Saito[2], Oleg Domanov[3], Hidetsugu Shiozawa[3,4], Sándor Lenk[5], Béla Pécz[6], Pál Koppa[5] and Katalin Kamarás[1]

[1]Institute for Solid State Physics and Optics, Wigner Research Centre for Physics, Hungarian Academy of Sciences, Konkoly Thege u. 29-33, 1121 Budapest, Hungary

[2]Nanomaterials Research Institute, AIST, 1-1-1 Higashi, Tsukuba 305-8565, Japan

[3]Faculty of Physics, University of Vienna, Boltzmanngasse 5, A-1090 Vienna, Austria

[4]J. Heyrovsky Institute of Physical Chemistry, Academy of Sciences of the Czech Republic, Dolejskova 3, CZ-182 23 Prague 8, Czech Republic

[5]Department of Atomic Physics, Budapest University of Technology and Economics, Budafoki út 8, H-1111 Budapest, Hungary

[6]Institute for Technical Physics and Materials Science, Centre for Energy Research, Hungarian Academy of Sciences, Konkoly Thege u. 29-33, 1121 Budapest, Hungary

Sample Preparation Methods

- For Optical measurements

We used a vacuum filtration technique in order to place very small amounts of nanotube onto a silicon substrate. As a first step a small piece from the buckypaper of nanotubes was dispersed in aqueous solution of Triton X-100 surfactant followed by 2h of sonication to reduce the size of the bundles. The suspension was then further diluted until one could only barely perceive the grayish tint of the solution.

From this solution 125 μL was filtered onto an acetone soluble filter membrane. The membrane was transferred onto a Si surface and was dissolved in boiling acetone. The CNT-Si samples were then washed by ethanol and isopropyl alcohol.

After the optical measurements on the as-prepared samples we applied the annealing process (2h at 700 °C in vacuum) on the same CNT-Si sample to create Ni clusters.

- For TEM measurements

For the TEM measurements we annealed (2h at 700 °C in vacuum) a small buckypaper nanotube piece. After the annealing process we sonicated it in toluene for 1h. Nanotubes were transferred onto lacey carbon TEM Grid.

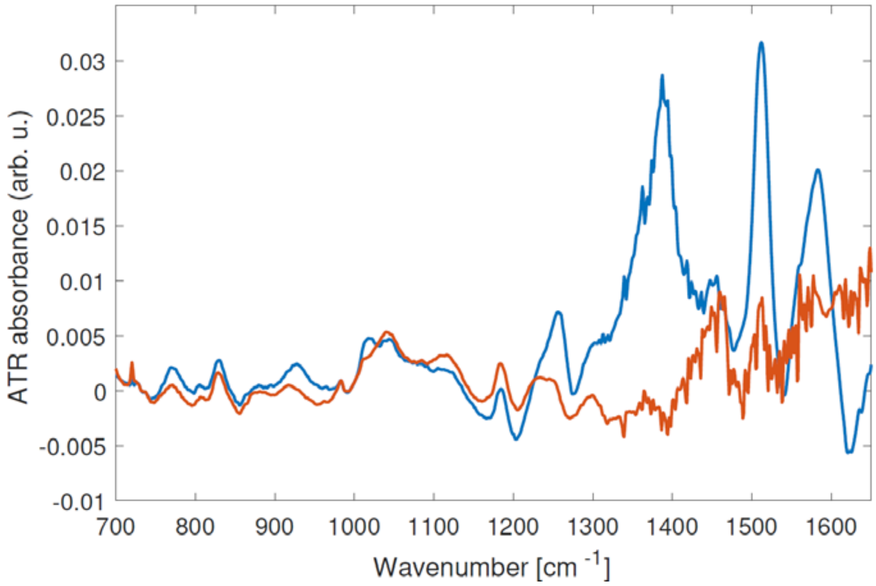

Figure S1: ATR-IR spectra of Ni-acetylacetonate-filled carbon nanotube acquired from the buckypaper sample, before (blue curve) and after (red curve) annealing. The absorption peaks of the molecules disappear as they dissociate and the remaining nickel forms clusters.

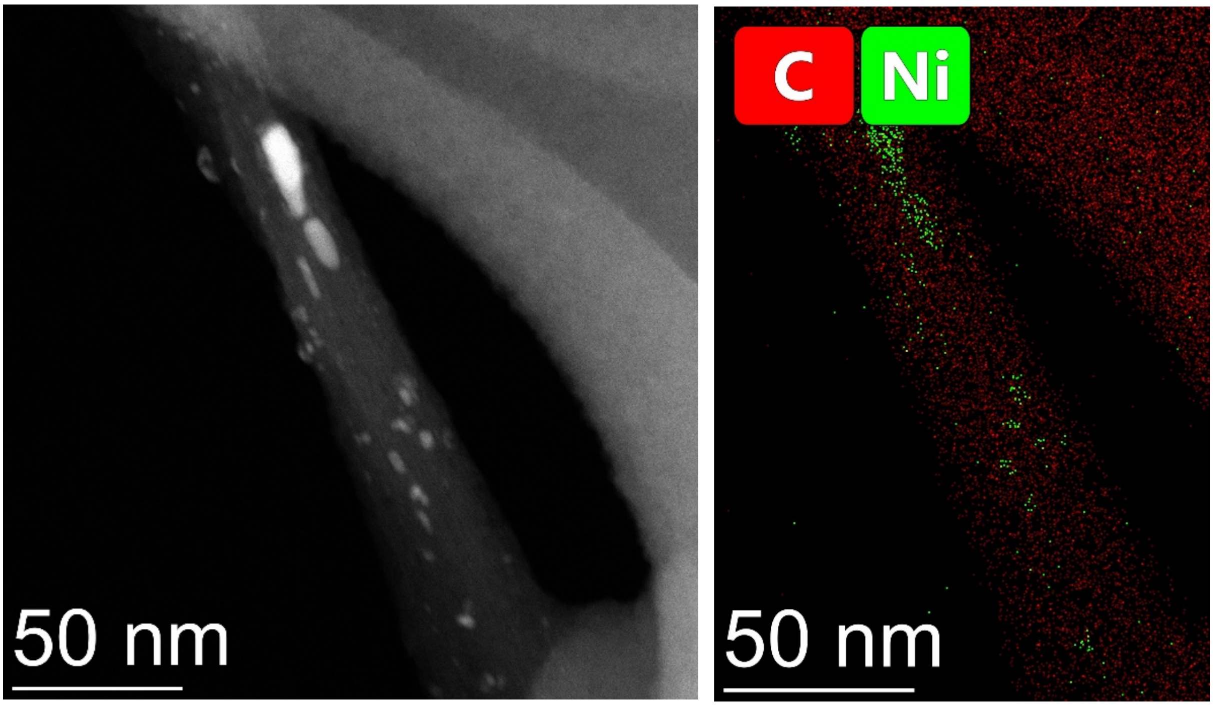

Figure S2: STEM-HAADF (High-angle annular dark-field) [Left] and EDS (energy dispersive X-ray spectroscopy) [Right] image of carbon nanotube bundles after high temperature annealing. The nickel clusters grown inside the nanotubes can be seen as bright spots on the HAADF image. The EDS map also verifies that those clusters contain Ni.

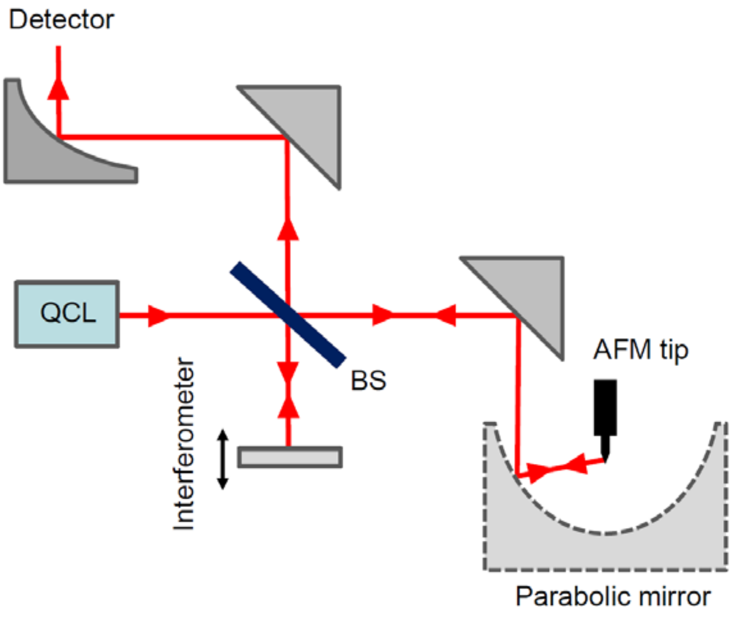

Figure S3 : Schematic presentation of the s-SNOM setup.

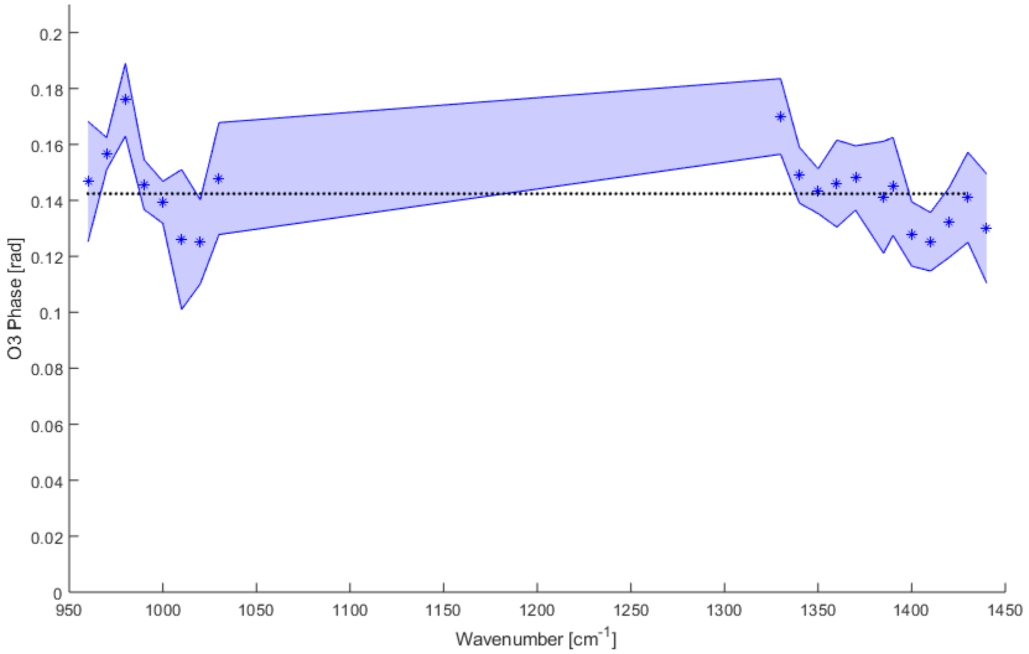

Figure S4 : O3 near-field phase spectrum of a nickel cluster formed inside a single-walled carbon nanotube. The black dotted line represents the phase calculated via EFDM. The blue stars show the measured phase at different wavenumbers in our tuning range. The blue area represents the measurement error range.

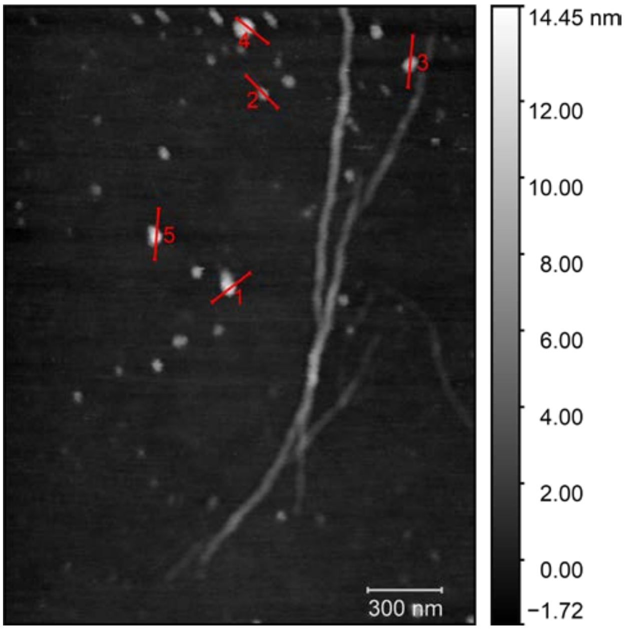

Figure S5 : AFM topography of Fig. 6 of the main text. Additional spots which appear also in contrast are measurement artifacts from dirt on the substrate.

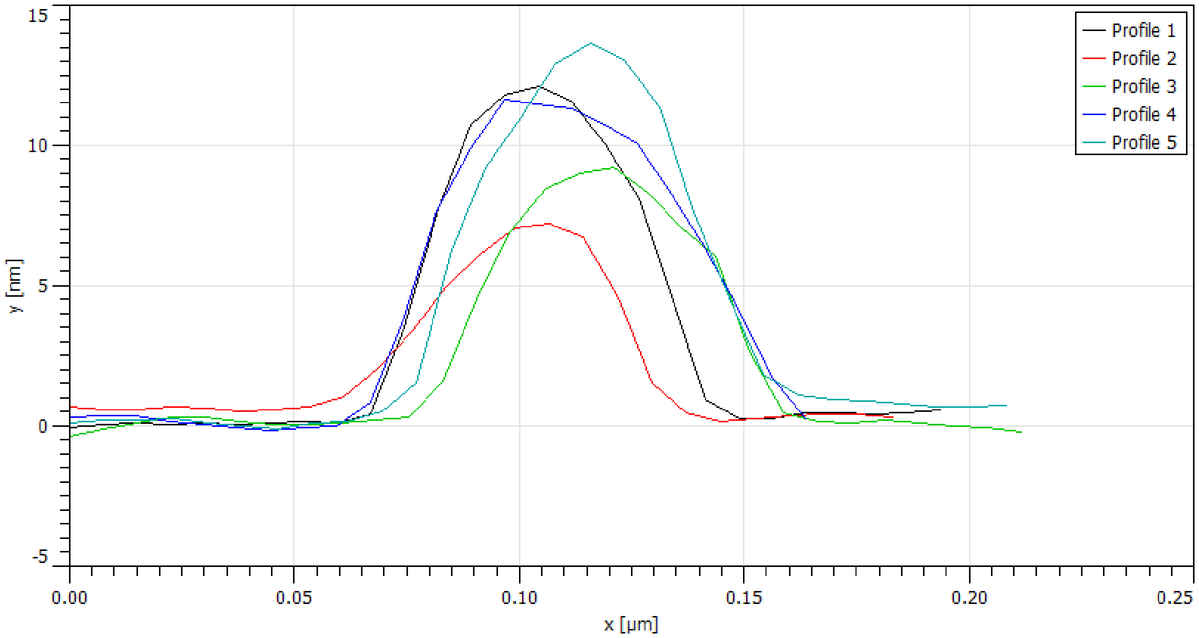

Figure S6: AFM topography profile of the spots mentioned in Fig. S5. These objects are high and provide low signal level which results in elevated noise in those areas and looks like signal contrast in low resolution images.
